# Polymerase-free measurement of microRNA-122 with single base specificity using single molecule arrays: Detection of drug-induced liver injury

**DOI:** 10.1371/journal.pone.0179669

**Published:** 2017-07-05

**Authors:** David M. Rissin, Barbara López-Longarela, Salvatore Pernagallo, Hugh Ilyine, A. D. Bastiaan Vliegenthart, James W. Dear, Juan J. Díaz-Mochón, David C. Duffy

**Affiliations:** 1 Quanterix Corporation, Lexington, Massachusetts, United States of America; 2 DestiNA Genomics Ltd., Edinburgh, United Kingdom; DestiNA Genomica S.L. Parque Tecnológico Ciencias de la Salud (PTS), Avenida de la Innovación 1, Edificio BIC, Armilla, Granada, Spain; 3 Edinburgh University/BHF Centre for Cardiovascular Science, The Queen's Medical Research Institute, Edinburgh, United Kingdom; 4 Pfizer-Universidad de Granada-Junta de Andalucía Centre for Genomics and Oncological Research (GENYO), Parque Tecnológico de Ciencias de la Salud (PTS), Avenida de la Ilustración 114, Granada, Spain; Universitat des Saarlandes, GERMANY

## Abstract

We have developed a single probe method for detecting microRNA from human serum using single molecule arrays, with sequence specificity down to a single base, and without the use of amplification by polymerases. An abasic peptide nucleic acid (PNA) probe—containing a reactive amine instead of a nucleotide at a specific position in the sequence—for detecting a microRNA was conjugated to superparamagnetic beads. These beads were incubated with a sample containing microRNA, a biotinylated reactive nucleobase—containing an aldehyde group—that was complementary to the missing base in the probe sequence, and a reducing agent. When a target molecule with an exact match in sequence hybridized to the capture probe, the reactive nucleobase was covalently attached to the backbone of the probe by a dynamic covalent chemical reaction. Single molecules of the biotin-labeled probe were then labeled with streptavidin-β-galactosidase (SβG), the beads were resuspended in a fluorogenic enzyme substrate, loaded into an array of femtoliter wells, and sealed with oil. The array was imaged fluorescently to determine which beads were associated with single enzymes, and the average number of enzymes per bead was determined. The assay had a limit of detection of 500 fM, approximately 500 times more sensitive than a corresponding analog bead-based assay, with target specificity down to a single base mis-match. This assay was used to measure microRNA-122 (miR-122)—an established biomarker of liver toxicity—extracted from the serum of patients who had acute liver injury due to acetaminophen, and control healthy patients. All patients with liver injury had higher levels of miR-122 in their serum compared to controls, and the concentrations measured correlated well with those determined using RT-qPCR. This approach allows rapid quantification of circulating microRNA with single-based specificity and a limit of quantification suitable for clinical use.

## Introduction

The sensitive detection of specific sequences of nucleic acids (NA) has become an indispensable tool in biological research, and in the diagnosis and treatment of diseases. The field of molecular diagnostics—where detection of specific sequences allows diagnosis of cancer, infectious diseases, and hereditary disease—has emerged from these technologies.[1,2] In recent years, the use and measurement of short sequences (<100 bases) of RNA, in particular, has proven useful for greater understanding and control of biological systems.[3,4] For examples: circulating microRNA (miRNA) (~22 bases) regulates gene expression and is proposed as a diagnostic biomarker for several cancers and others pathologies; interfering RNA has been used as a therapeutic via gene silencing; and, RNA probes are the basis of gene editing techniques, such as CRISPR/Cas9. The polymerase chain reaction (PCR) and next generation sequencing (NGS) technologies dominate the detection of NA.[1] These methods have some limitations in detecting NA, in particular short strands of RNA. Detection of short sequences is challenging for assays that require multiple binding molecules, e.g., primers of PCR or sandwich binding assays. This requirement often necessitates the elongation of the target molecule using either a ligation step with an extension sequence so that the probes can bind, or use of poly(A) polymerase to poly-adenylated target NA, so that they can be converted into cDNA (as in the RT-qPCR assay used here). PCR also suffers from bias, sample contamination from production of high concentration of amplicon, and requires laborious sample preparation.

Measurement of miRNA, which in humans and rodents are around only 22 nucleotides in length, has presented unique analytical challenges when requiring highly precise and accurate quantification for diagnostic proposes. The diagnostic and prognostic value of miRNAs as biomarkers, however, has been recognized in many thousands of scientific publications. In particular, microRNA-122 (miR-122) has been shown to be a more sensitive and specific biomarker for liver toxicity when compared with standard protein biomarkers used to date.[5,6] Techniques that address the challenges of existing assays for NA, especially for short sequences of RNA, but maintain the high analytical sensitivity and sequence specificity of PCR are needed. For example, the Deveraj group recently reported a method for detecting miRNA-21 based on turnover amplification of the fluorogenic response in nucleic acid-templated reactions.[7] The method was able to detect 500 fM of miR-21 that was specific to a single-base mismatch, but no comparison to PCR or clinical application was provided.

Here, we report a method for detecting short sequences of RNA based on the hybridization of single native RNA molecules using a single immobilized probe, without the use of amplification using polymerases. The approach combines the specific labeling of an immobilized capture probe with a biotinylated single nucleobase, through the templating action of target RNA molecules, with detection in arrays of femtoliter wells of single enzymes that bind to the biotin. We have previously described the use of the dynamic labeling chemistry to specifically label NA probes with a hapten-presenting base, when the probe was hybridized to the complementary target sequence.[8] As we [8] and others [9] have reported, nucleic acid analysis by dynamic chemistry harnesses Watson–Crick base pairing to template a dynamic reaction on a strand of an abasic peptide nucleic acid [PNA; a DNA mimic in which the sugar-phosphate backbone is replaced with N-(2-aminoethyl) glycine]. The dynamic reaction is achieved by hybridizing an abasic PNA probe to the target nucleic acid strand such that a nucleobase-free position on the PNA (a so-called “blank” position) lies opposite to a nucleotide on the target nucleic acid strand. The reversible reaction between an aldehyde-modified nucleobase and a free secondary amine on the PNA probe generates an iminium intermediate that can be reduced to a stable tertiary amine by a reducing agent. Different iminium species can be generated, but the one with the correct hydrogen bonding motif (obeying Watson–Crick base-pairing) will be the most thermodynamically stable product. Complementary nucleic acid strands also act as catalysts to accelerate the rate of reductive amination; when there is not complementary nucleic strands, reductive aminations do not happen within the assay timeframe, as we have shown previously.[8] Assay signals were then generated using specific hapten-binding reagents, allowing target quantification. This approach has been used to demonstrate genotyping assays using mass spectrometry,[8] DNA microarrays,[10] and conventional bead-based assays.[11] The sensitivity of these analog assays were, however, not sufficient to detect miRNA in clinical samples.[11] We have also demonstrated that single molecule arrays (Simoa)[12] are very sensitive to an enzyme label (limit of detection, LOD = 220 zM), and we have used this capability to develop sensitive assays for proteins[12] and DNA.[13] The Simoa DNA assay was, however, limited to relatively long target sequences (>100 base pairs) because of the requirement for a capture and multiple detection probes, each being 15–20 bases long. Combining the dynamic chemistry and single molecule array approaches has enabled an assay with single label sensitivity and single base specificity using a single, short (<20 base) probe for detecting short RNA target molecules. We applied this method to detect miR-122, a biomarker of liver toxicity, in serum.

## Materials and methods

### Ethics statement

The collection of samples from human participants in this study was approved by the authors' committee equivalent to the Institutional Review Board (IRB), and all clinical investigation was conducted according to the principles expressed in the Declaration of Helsinki. Informed consent, written or oral, was obtained from the participants. For patients with liver injury, full informed consent was obtained from every participant and ethical approval for this study was from the South-East Scotland Research Ethics Committee. For healthy volunteers, the study was approved by the local research ethics committee (East Midlands–Nottingham 1 Research Ethics Committee), and performed in accordance with the Declaration of Helsinki. Informed consent was obtained from all participants.

### Materials

RNA target molecules were purchased desalted from Integrated DNA Technologies. All chemicals were obtained from Sigma Aldrich, and used as received. 2.8-μm diameter superparamagnetic beads presenting carboxylic acid groups (Dynabeads^®^ M-270), and resorufin-β-D-galactopyranoside (RGP) were obtained from ThermoFisher Scientific. Streptavidin-β-galactosidase (SβG) was conjugated in house using methods described previously.[12] Simoa disks comprised of 24 arrays of 238,764 50-fL-sized microwells molded into cyclic olefin copolymer (COC) and bonded to a microfluidic manifold were obtained from Stratec Consumables.[14] Fluorocarbon oil (Krytox^®^) was obtained from DuPont. Concentrations of solutions of RNA and DNA were determined using a ThermoFisher NanoDrop1000 Spectrophotometer.

### Synthesis of capture probe and aldehyde-modified, biotinylated nucleobase

A PNA probe containing an abasic “blank” position and terminated with an amino-PEG linker was synthesized using standard solid phase chemistry on a MultiPep Synthesiser (Intavis AG GmbH, Germany). The sequence of the probe (**1**) was designed to allow anti-parallel hybridization with the mature miR-122 target (**2**); sequences are shown in Table 1. The probe contained 3 thymidine bases modified with propionic acid side chains to increase its negative charge.[11] The structure of the amino-PEG linker is shown in S1 Fig. Aldehyde-modified cytosine, tagged with biotin via a 12 ethylene glycol units spacer was prepared using a synthetic route described elsewhere.[8]

**Table 1:** Sequences of capture probe (1) and miR-122 target (2).

ID	Name	Peptide with abasic position (Nˈ-Cˈ)
**1**	Capture probe	xx-CACCAT*TGT*_ACACT*CCA
		**miRNA sequence (5ˈ-3ˈ)**
**2**	Target miR-122	*UGGAGUGU**G**ACAAUGGUG*UUUG

*Key*: xx = amino-PEG-linker (S1 Fig); T* = thymidine containing a propanoic acid side chain at the gamma position; “_” = abasic “blank” monomer presenting a secondary amine; the italicized bases in **2** form a duplex with the capture probe, and G at position 9 (bold) is opposite the “blank” monomer and binds to the aldehyde-modified cytosine. The mature sequence of miR-122 is 22 bases long. Our probe targets 18 bases out of the 22 (italics), leaving out the 4 bases at the 3’-end.

### Preparation of superparamagnetic beads presenting probes

100 μL of superparamagnetic beads (containing 2 × 10^8^ beads) were washed by adding 100 μL 0.01 M NaOH and mixing. The beads were pelleted, supernatant removed, and the beads were washed once in 100 μL 0.01 M NaOH and three times in 100 μL distilled water. The beads were then resuspended in 150 μL of freshly-prepared 50 mg/mL 1-ethyl-3-(3-dimethylaminopropyl)carbodiimide (EDC) in water, and incubated with slow tilt rotation at 23°C for 30 min. After activation with EDC, the beads were washed once with 100 μL cold water and once with 100 μL cold MES buffer (50 mM, pH 5.0). 100 μL of a solution containing 1400 pmol capture probe (**1** in Table 1) and 700 pM of an amino-terminated diluent DNA sequence (5ˈH_2_N-GAGTGTTAGCTGTGAG 3ˈ) in MES buffer (50 mM, pH 5) was added to the activated beads. The mixture of probe and beads was incubated at 4°C for 3 h with slow tilt rotation, and then washed with 50 mM MES buffer (pH 5). The amount of probe that coupled to the bead (~600,000 probes/bead) was measured using UV to ensure lot-to-lot reproducibility. The remaining activated carboxyl groups were quenched by incubating the beads with 100 μL of 50 mM ethanolamine in PBS (pH 8) for 1 h, followed by three washes with 100 μL of 10% PEG10K and 0.1% Tween-20 in PBS. The capture beads were stored at 4°C in 100 μL of 10% PEG10K and 0.1% Tween-20 in PBS.

### Collection and preparation of clinical samples

*A) Samples from patients with drug-induced liver injury*. Samples from 4 patients (Patient Identifiers 3–4, 6, and 9) with acetaminophen (APAP) induced liver injury were recruited as part of the Markers and Paracetamol Poisoning Study (MAPP). Adult patients (16 years old and over) were recruited to the MAPP study if they fulfilled the study inclusion and exclusion criteria. Full informed consent was obtained from every participant and ethical approval for this study was from the South-East Scotland Research Ethics Committee. The inclusion criteria were: a history of APAP overdose that the treating clinician judged to warrant treatment with intravenous acetylcysteine as per the contemporaneous UK guidelines; the first blood sample collected within 24 h of last APAP ingestion; and, the patient has capacity to consent. Patients were excluded if any of the following applied: patient detained under the Mental Health Act (UK); patient has known cognitive impairment; inability to provide informed consent for any reason; or an unreliable history of overdose. Patients having taken a single acute APAP overdose were recruited at the Royal Infirmary of Edinburgh, UK. The demographic details and clinical chemistry measurements performed on the patients are shown in S1 Table. *B) Healthy volunteers*. The study was approved by the local research ethics committee (East Midlands–Nottingham 1 Research Ethics Committee), and performed in accordance with the Declaration of Helsinki. Informed consent was obtained from all participants. A total of 4 adults (23–42 years old) were recruited to this study (Patient identifiers HV1-4). Healthy volunteers were eligible if they had no history of liver disease, they were taking no medications, and they were willing to give blood samples by venepuncture.

For both healthy volunteers and patients with liver injury, blood samples were centrifuged immediately at 11,000 × *g* for 15 min at 4°C, after which serum was separated into aliquots and frozen at −80°C.

### Preparation of samples spiked with known concentrations of a synthetic calibrator for miR-122

Serum from a healthy donor, who was negative for acute liver failure, was used for spike-in experiments. The blood from the donor was centrifuged at 11,000 × *g* for 15 min at 4°C after which serum was separated into 6 aliquots. A synthetic calibrator for miR-122 (**A**, S2 Table) at different concentrations were added to each aliquot to yield 6 control samples (Control samples 1 to 6 containing: 10 nM, 10 nM, 1 nM, 1 nM, 100 pM, and 10 pM, respectively). These samples were frozen at −80°C and subsequently processed in an identical fashion as the clinical samples.

### Isolation of RNA from serum samples

RNA was isolated from serum using the miRNeasy Serum/Plasma kit (Qiagen) according to manufacturer’s protocol. Briefly, 500 μL QIAzol lysis reagent was added to 100 μL thawed serum, mixed, and incubated at 23°C for 5 min. To calibrate the concentration of miR-122 and as a control to determine the efficiency of recovery and reverse transcription of miRNA, 3.5 μL of cel-miR-39-3p (1.6 × 10^8^ copies/μL) was added to each sample, in addition to 100 μL chloroform. Following shaking, incubation, and centrifugation, the upper aqueous phase was transferred, and 450 μL of ethanol was added and transferred to the RNeasy MinElute column. The column was washed with buffers RWT and RPE from the miRNeasy kit, and 80% ethanol, followed by drying and elution in 14 μL RNase-free water; total miRNA was recovered in ~12 μL of water ([cel-miR-39-3p] = 4 × 10^7^ copies/μL). Quantification and quality assessment of small RNA, including the miRNA fraction, were performed using the Small RNA Assay kit (Agilent Technologies). Purified RNA was stored at < −80°C before analysis with Simoa and RT-qPCR.

### Determination of concentration of miR-122 using quantitative real time PCR

PCR was performed using a leading commercial kit (Qiagen) and a procedure that has been described in detail elsewhere.[15] cDNA was generated by reverse transcription using the miScript RT II kit (Qiagen) on 1.5 μL of RNA purified from serum (reaction volume = 20 μL). This step includes the use of a poly(A) polymerase to poly-adenylated the target miRNAs, so that the molecule was sufficiently long to be converted into cDNA. 200 μL of RNase-free water was added to the sample containing cDNA, and 1 μL of this solution ([cel-miR-39-3p] = 2.7 × 10^5^ copies/μL) was added to the PCR reaction mixture (reaction volume = 25 μL). Quantitative PCR analysis was performed using the miScript SYBR Green PCR Kit (Qiagen) on a 7900HT Fast Real-Time PCR System (Applied Biosystems, CA), with an initial activation step of 95°C for 15 min followed by 40 cycles of 3-step cycling (Denaturation: 15 s at 94°C; Annealing: 30 s at 55°C; Extension: 30 s at 70°C). Ct values were determined for miR-122 and miR-39 in separate PCR reactions.

A calibration curve of Ct as a function of miRNA was determined by performing PCR on miR-39 at 5 ×10^5^, 5 ×10^4^, 5 ×10^3^, and 5 ×10^2^ copies/μL (independent from serum samples) according to manufacturer’s protocol (miRNeasy Serum/Plasma Spiked-In Control, Qiagen). This calibration curve was used to determine the recovery of miR-39 from the samples based on the 10,800 copies/μL spiked control in the final PCR reaction. The calibration curve was also used to determine the concentration of miR-122 in each sample following the protocol reported in the miRNeasy Serum/Plasma Handbook. Concentrations of miR-122 were also corrected for the recovery measured for miR-39.

### Determination of concentration of miR-122 using Simoa

*1) Sample preparation (manual)*. Sample hybridization and labeling of the capture probe on the beads was conducted in a 96-well conical bottom microtiter plate (ThermoFisher Scientific; Cat no. 249944). The total volume of the labeling reaction was 50 μL, containing 7.5 μL of sample or calibrator, 4,000,000 capture probe beads, 5 μM aldehyde-modified biotinylated cytosine, 1 mM sodium cyanoborohydride, 0.1% SDS, and 10% w/v PEG-10K, in a buffered solution of 30 mM trisodium citrate and 300 mM sodium chloride, pH adjusted to 6.0 using HCl. The microtiter plate was placed on an incubating shaker (VWR; Cat no. 12620–930), set to 40°C, and mixed at 1000 rpm for 1 h. The beads were then pelleted on a custom-made 96-well plate magnet (VP Scientific), and washed 3 times with 230 μL of PBS and 0.1% Tween 20, followed by resuspension of the beads in 230 μL of PBS and 0.1% Tween 20. Manual sample preparation of 24 samples, including preparation of reagents, calibrators, and samples, took 135 min. *2) Enzyme labeling step and quantification using Simoa (automated)*. The beads were analyzed on a Simoa HD-1 Analyzer (Quanterix Corporation).[16] The microtiter plate containing the labeled beads were loaded onto the instrument, along with enzyme conjugate and enzyme substrate (SβG and RGP, respectively), and consumables (Simoa disks, pipette tips, and reaction cuvettes). The Simoa measurements occurred in two steps on this instrument: liquid handling for enzyme labeling of the biotin-labeled probe beads; and, imaging and image analysis of the beads sealed in the Simoa disk. 100 μL of the solution containing beads in the microtiter plate was pipetted from the well into a reaction cuvette by a disposable tip pipettor. 100 μL of a solution containing 500 pM of SβG was added by a fixed tip pipettor to the reaction cuvette, the cuvette was shaken to disperse the beads, and incubated for 5 min. The beads were then magnetically separated and washed six times in 5× PBS and 0.1% Tween 20, and washed once in PBS. After aspiration of PBS, 25 μL of 100 μM RGP in PBS was added by a disposable tip pipettor to the reaction cuvette, the beads were mixed, and 15 μL of this bead-substrate mixture was transferred into an inlet port of an array on a Simoa disk. The beads were pulled by vacuum over the array of femtoliter wells, and the beads settled into the array of femtoliter wells. The beads were sealed with oil, and the single enzyme signal was generated over 30 s. The arrays were fluorescently imaged at multiple wavelengths at submicron resolution as described previously.[16] These fluorescent images were used to identify the location of beads within the wells, and the enzyme activity associated with each bead. Image analysis software on the instrument determined the average number of enzymes per bead (AEB).[17] From the resulting AEB values of calibrators of known concentration, the concentrations of miR-122 in samples of unknown concentration were determined from interpolation using linear curve fitting in Microsoft Excel. Each sample was analyzed in duplicate to provide a mean AEB and a standard deviation. Simoa analysis of 24 samples, including instrument set up and run time, took 75 min, resulting in a total assay time of 3.5 h.

## Results and discussion

**Fig 1:**
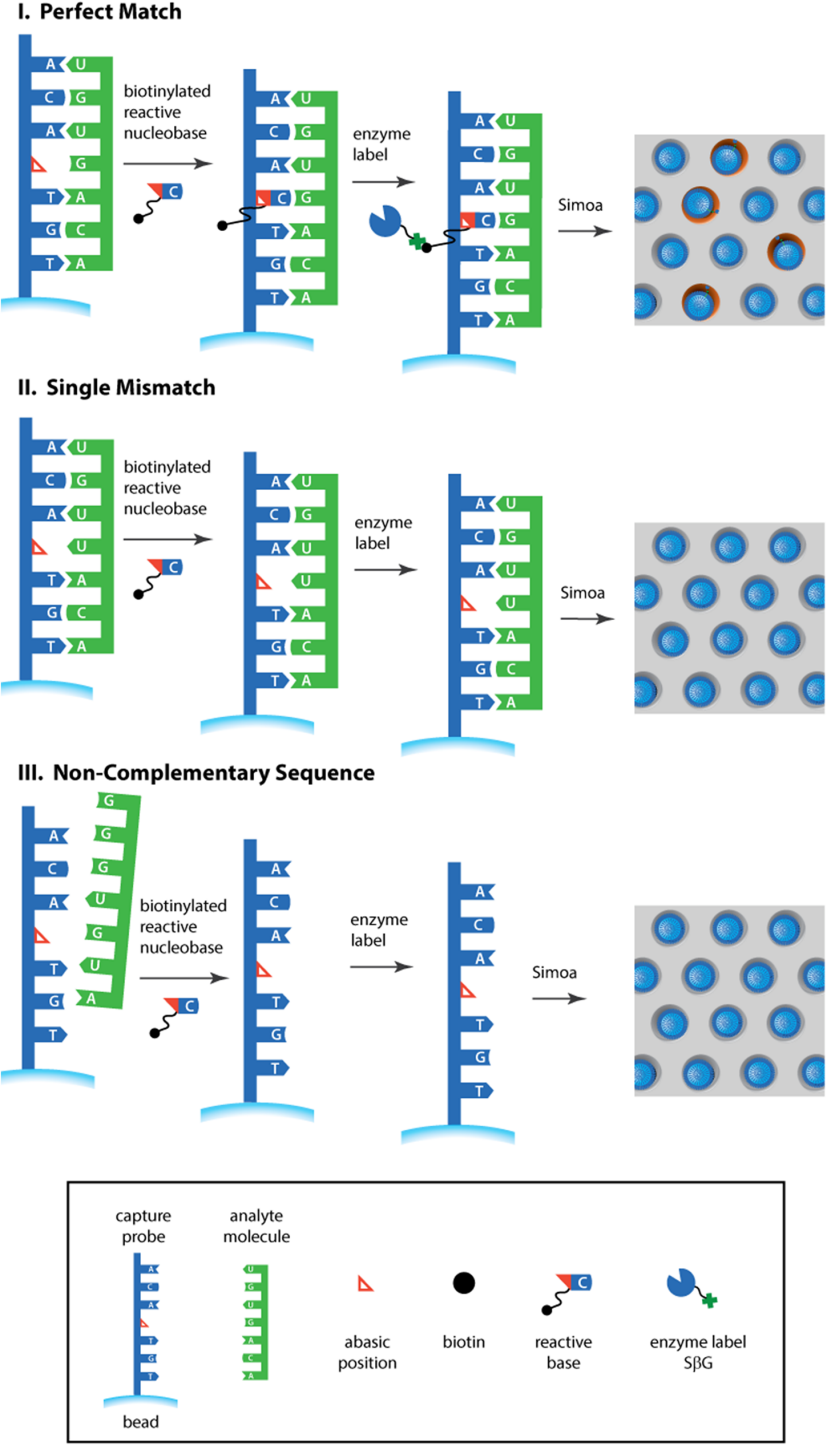
Schematic of the assay for miRNA based on combining dynamic labeling of specific single bases and detection using single molecule arrays.

**Fig 2:**
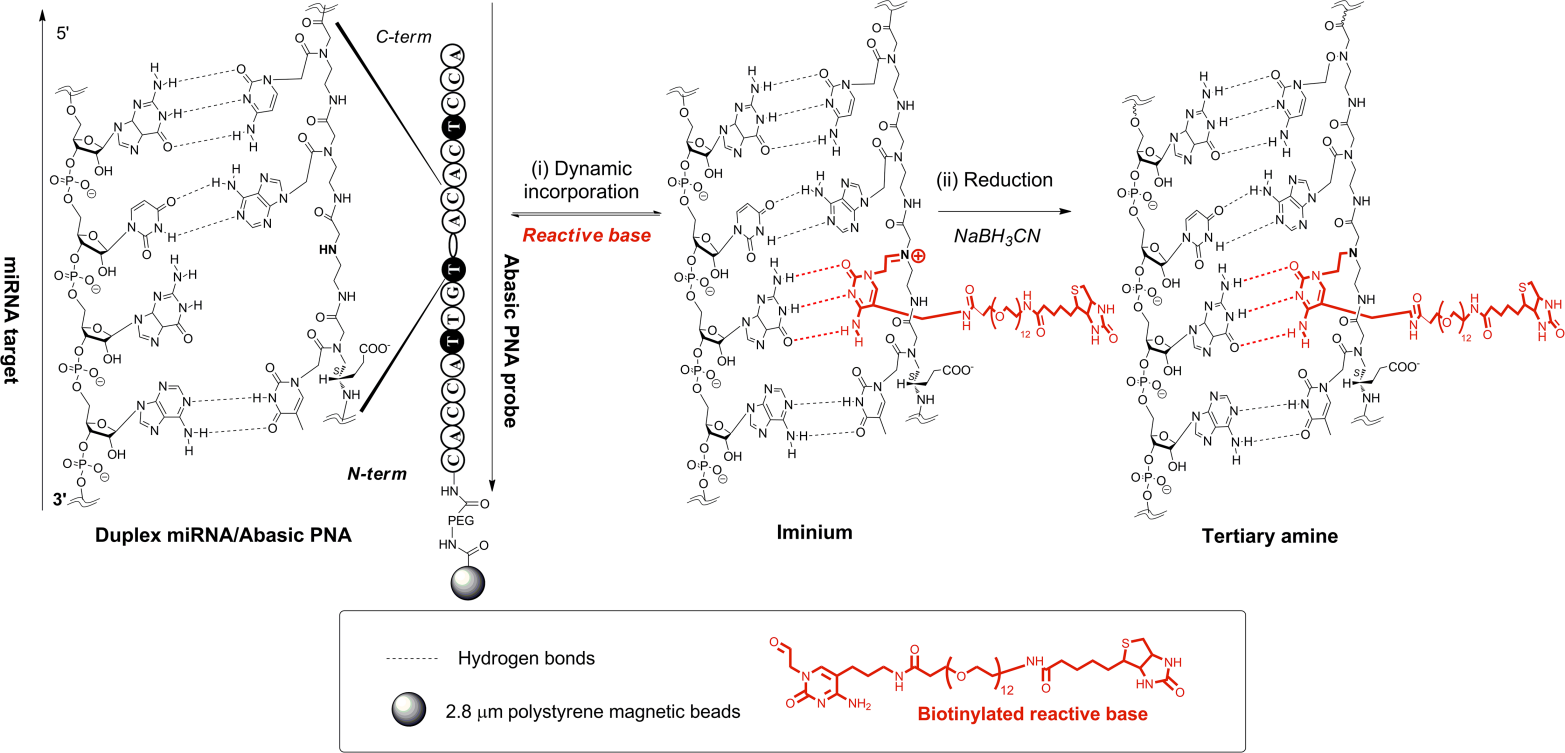
Dynamic chemistry labeling of biotin into an immobilized abasic PNA probe on a superparamagnetic bead.

Fig 1 shows a schematic of our approach to detecting single molecules of miRNA with single base specificity; Fig 2 shows the steps of the dynamic chemistry reaction that was used to incorporate biotin into the immobilized probe. A detailed description of the preparation of reagents and assay steps for detecting miR-122 are provided in the Materials and Methods section; a brief description follows here. A specific 18-mer abasic PNA probe that was complementary to miR-122 was synthesized with the cytosine base at the 9^th^ position from the C-terminus of the PNA probe replaced with a secondary amine group to yield a “blank” position in the capture sequence. Previous reports have examined the optimal position of the blank base in the abasic probe:[8,9] in general, the best positions for the blank base are located towards the middle of the abasic probe. Different probe designs can be synthesized depending on the selected base to be interrogated and the target position on the probe. Here, we elected to use an aldehyde-modified cytosine to interrogate a guanidine residue on the target miR-122 (Table 1). 3 thymidine bases in the PNA probe were modified with propionic acid at their gamma positions to improve hybridization efficiency,[11] and the N-terminus of the probe was a primary amine. The sequence of the probe (**1**) is shown in Table 1. The probe was covalently attached via its N-terminal amine to superparamagnetic beads presenting surface carboxyl groups.

These beads were then incubated with a solution containing the sample, an aldehyde-modified cytosine base that contains biotin (Fig 2), and a reducing agent, in reaction buffer with a total volume of 50 μL. If miR-122 was present in the sample then it hybridized to the complementary capture probe on the beads (**I,** Fig 1). As the target sequence (**2** in Table 1) has a guanine at the 9^th^ position from the 5’-end that lines up with the “blank” position in the capture probe, the cytosine base binds to this guanine, and places the aldehyde in close proximity to the secondary amine on the probe backbone. The aldehyde and amine reacted to form a thermodynamically stable iminium complex that was then reduced to a stable tertiary amine by a reducing agent, thereby covalently incorporating biotin into the probe attached to the beads (Fig 2). Molecules with the same sequence but a single base mis-match at the 9^th^ position, hybridize but a stable iminium group did not form and biotin was not incorporated (**II**, Fig 1). Non-complementary sequences do not hybridize to the capture probes and biotin was not incorporated (**III**, Fig 1). In summary, this approach requires two specific molecular events to create a signal: (i) perfect hybridization between nucleic acid strands and the abasic PNA probe; and, (ii) specific molecular recognition, through Watson-Crick base-pairing rules, by the modified nucleobase.

After labeling of the immobilized probe, the incorporated biotin labels were then labeled with an enzyme (SβG). At low concentrations of miR-122, the ratio of enzyme labels to beads was <1, so that the distribution of enzymes on the beads followed a Poisson distribution,[12] and single miR-122 molecules were labeled. These enzyme-labeled miR-122 molecules were detected by loading the beads into arrays of ~239,000 microwells in the presence of a fluorogenic substrate of β-galactosidase (RGP). The wells were sealed with oil, so that the product of the enzyme-substrate reaction was confined to a small volume (~ 50 fL). Single beads and associated enzyme activity in the wells were measured using a fluorescent imager with sub-micron resolution. The fraction of active beads and average number of enzymes per bead (AEB) were then determined as described previously.[17] In summary, the assay comprised a duplex formation between the target miRNA and its complementary abasic PNA probe, into which the aldehyde-modified biotinylated base was covalently bound to the probe, itself attached to a magnetic bead. The biotinylated bead was then recognized by SβG that generated fluorescent signals from single beads. The signal was proportional to the concentration of target miRNA.

**Fig 3:**
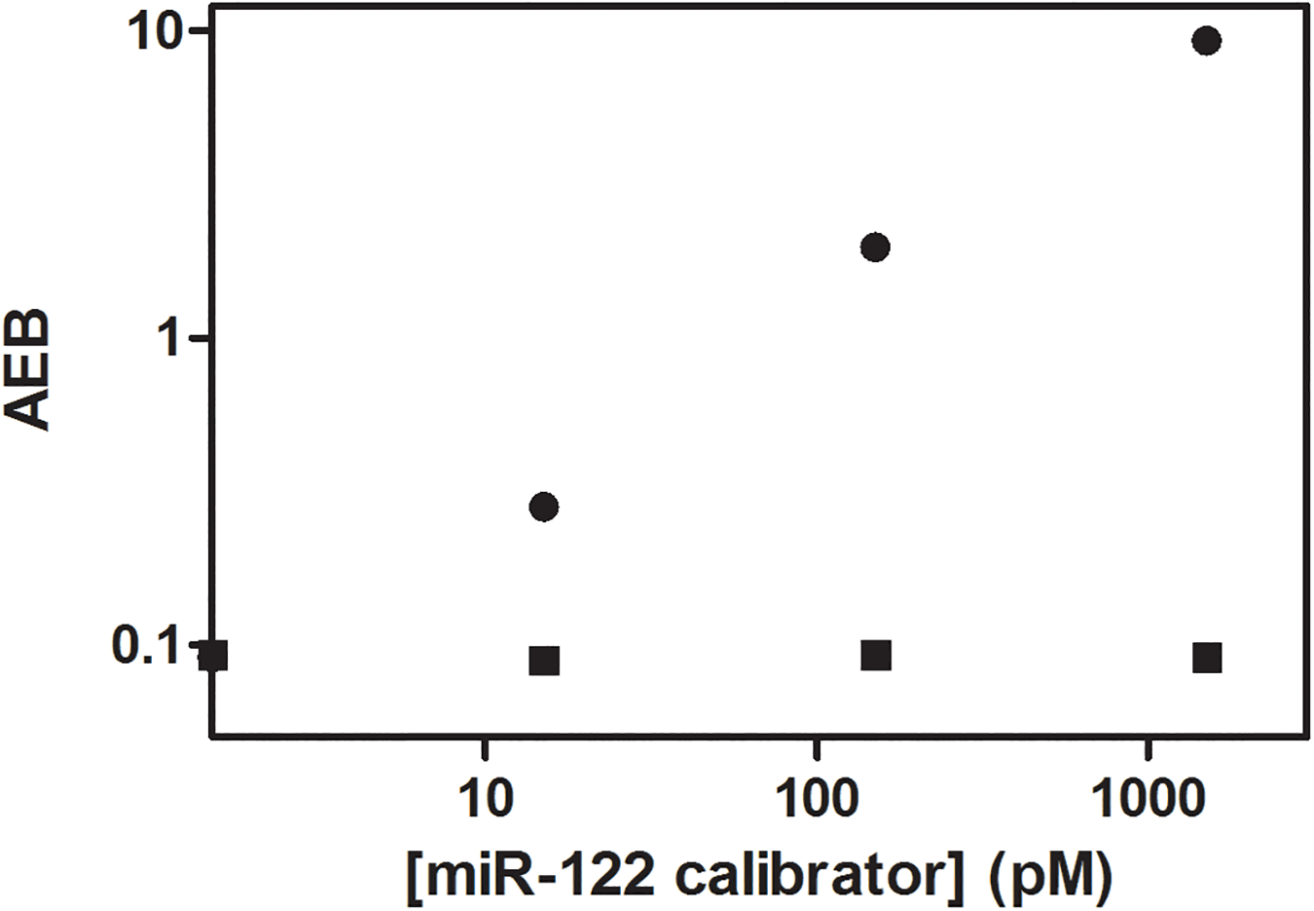
Plot of AEB determined using the assay in Fig 1 against concentration of calibrators for miR-122 (circles) and the same molecule with a single base mismatch at the 9^th^ position (squares) spiked into buffer. The sequences of these two molecules are shown in S2 Table. Error bars (±1 s.d.) based on duplicate measurements are smaller than the size of the data point.

Using this approach, we evaluated the analytical sensitivity of the assay for measuring miR-122, and compared it to a PCR kit that is used widely to measure microRNA.[15] miR-122 is a known biomarker of drug-induced liver toxicity that is more sensitive and specific than conventional markers of liver toxicity.[18] The US Food and Drug Administration (FDA) and the European Medicines Agency support its use in drug development. Fig 3 shows AEB values for buffered solutions into which known concentrations of a synthetic calibrator for miR-122 were spiked ranging from 0 to 1500 pM. The limit of detection (LOD) of the Simoa assay was 500 fM, based on interpolation of the concentration at 3 s.d. of the background above background. The LOD for the same molecule using a conventional, analog bead-based assay was 300 pM, as previously reported.[11] The LOD for a commercial RT-qPCR assay for miR-122 was 2.6 fM (S3 Table and S2 Fig).

The greatly improved sensitivity (600-fold) of the single molecule assay over the equivalent analog assay was sufficient to detect miR-122 precisely in the clinical samples from the patients being examined here (see below). There is also considerable scope for improving the sensitivity of this Simoa assay to approach that of PCR. The sensitivity of Simoa assays are a trade-off between the ability to efficiently capture and label target molecules (increasing signal), and increases in non-specific binding of enzyme conjugate to the bead associated with using more labeling reagents (reactive nucleobase and reducing agent), as has been described previously.[12] We typically optimize the sensitivity of Simoa assays by maximizing the capture of target molecules, and then use the minimum concentrations of labeling reagents to yield the highest signal-to-background ratio. For the application studied here, it was not necessary to systematically maximize the efficiency of target capture, so we used higher amounts of labeling reagents than is optimal to achieve sufficient sensitivity. As a result, the sensitivity of the assay was not optimized and was less than the PCR assay. We have a high degree of confidence that further optimization would result in a much more sensitive Simoa assay for several reasons. First, Simoa is very sensitive to enzyme label associated with beads—we have shown that the technique can detect 220 zM of SβG [12]—so the method has high intrinsic sensitivity to draw upon during assay optimization. Second, we have previously used this high label sensitivity to develop bead-based assays that can detect subfemtomolar concentrations of proteins [16] (e.g., 0.6 fM of PSA in serum),[12] and 0.07 fM of DNA [13] using similar approaches, albeit with different reagents. Third, the overall efficiency of the un-optimized process described here is low (number of molecules captured and labeled ÷ total number of molecules in the sample = 0.08%), indicating that there are significant opportunities to optimize capture efficiency of target while minimizing non-specific interactions of labeling reagents with beads. Our strategy in the future when greater sensitivity is needed would be to exploit these opportunities to maximize target capture efficiency and labeling efficiency resulting in more sensitive assays. The specificity of the assay was determined using two nucleic acid molecules. First, a random, off-target miRNA molecule (*C*.*elegans* miR-39; sequence in S2 Table) was tested. S3 Fig shows AEB values of the off-target miRNA compared to the calibrator for miR-122; the AEB values for miR-39 were at background. Second, a molecule that differed in sequence from miR-122 by a single base at the complement to the labeling position was tested. Fig 3 shows the AEB values of samples in which a molecule that had a single base mismatch at the 9^th^ position was spiked over the same concentration range. AEB for these samples did not increase significantly over background signal. Concentrations up to 15 μM of the single based mismatched molecule were also tested to quantify the specificity of the assay (S4 Table), with no increase in signal above background. Based on these data, the analytical specificity of the assay to a single-base mismatch defined as (highest concentration of mismatched target below LOD)/(LOD) was >3 × 10^7^-fold. For comparison, we also examined the specificity of the PCR assay for miR-122 by comparing the Ct values of the target miRNA and the single base mismatched molecule across the concentration range (S4 Fig). These data show that the Ct values of miR-122 with a single base mismatch did not differ significantly from the perfect match target, indicating that conventional RT-qPCR does not have the specificity of the Simoa assay described here. Conventional RT-qPCR does not exhibit single base specificity, so specialized assays based on polymerase amplification using specific fluorogenic probes such as TaqMan probes have been developed to provide this specificity.[19] In terms of specificity to different forms of miRNA, we note that miRNA biogenesis can be regulated at multiple levels, including modification by RNA editing, RNA methylation, uridylation, adenylation and RNA decay.[20,6] The platform presented here provides opportunities to design assays that can probe different forms of miRNA at a single base level. Therefore, with development, this approach could facilitate the sensitive and specific quantification of miRNAs that have undergone *in vivo* sequence modification.

**Fig 4:**
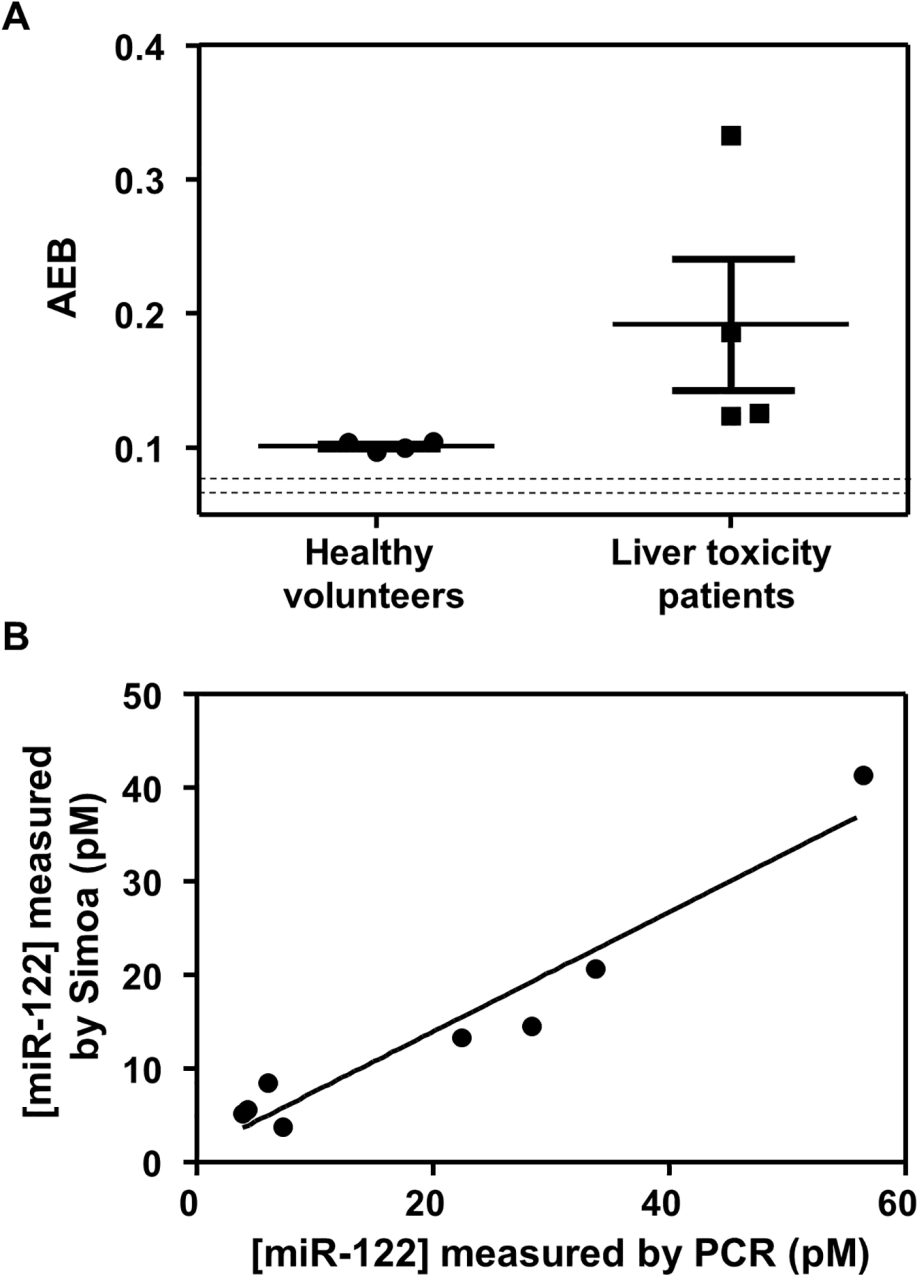
(A) Scatter plot of AEB for miR-122 measured in the serum of 4 healthy volunteers and 4 individuals after overdosing on acetaminophen. The dotted lines represent the mean assay (buffer) background ± 1 s.d.. (B) Correlation of concentration of miR-122 in the serum of patients determined using Simoa and PCR (r^2^ = 0.93; slope = 0.64).

Based on the sensitivity and specificity of the single molecule assay for miR-122, we measured miR-122 in the serum of patients who had overdosed on acetaminophen and sustained clinically significant liver injury, and in the serum of healthy individuals. The details of collection and processing of clinical samples are provided in the Materials and Methods section, and the clinical chemistry results for liver injury patients are presented in S1 Table. Acetaminophen is the most common cause of liver toxicity in the Western world, but current tools for patient stratification are inadequate.[21] Recently, however, it has been shown that the measurement of miR-122 at first presentation in hospital stratifies patients by their risk of liver injury with high sensitivity and specificity.[18] We purified total miRNA from serum samples and tested for miR-122 using the single molecule assay shown in Fig 1, and RT-qPCR. Fig 4A shows AEB values for miR-122 in the serum of patients with liver injury and healthy controls. The corresponding Ct values from RT-PCR are plotted in S5 Fig. These data indicate that the sensitivity and specificity of the Simoa assay was sufficient to measure miR-122 in all patients, and showed a clear distinction between healthy controls and those with clinical liver toxicity after drug overdose, as was the case for PCR. miR-122 is one of the most qualified human disease miRNA biomarkers, but its translation to clinical utility has been limited by a lack of a rapid and precise assay, and the challenges of the workflow of PCR. The data presented here suggest that the single molecule detection approach could allow measurement of miR-122 in emergency settings and in early phase drug development.

Fig 4B shows the correlation between the concentrations in serum from patients determined by Simoa and PCR. S5 Table and S6 Table show how concentrations were calculated for both methods for two serum samples from each patient. The correlation between the two methods was good, and the Bland-Altman plot (S6 Fig) demonstrates that miR-122 concentration as measured by the single molecule assay is lower than PCR with an average bias of 6.1 pM (95% limit of agreement = -8.5–20.8 pM); further optimization would be expected to reduce this bias. These data indicate that, when combined with the clinical sample data, the Simoa assay is suitable for measuring the miR-122 specifically and accurately compared to the gold standard method. The AEB values for miR-122 in healthy individuals, however, were similar and slightly above background (dotted line in Fig 4A), an observation that could be caused by differences in the matrix of the samples and calibrator solutions, rather than the presence of miR-122. We are, therefore, hesitant to categorically assign concentrations to these samples. The same limitation applies to the PCR data, where the Ct values of the healthy controls (S6 Table) are close to the background values (S3 Table). Measurement using Simoa and PCR of miR-122 in healthy individuals could be explored further by purifying RNA from a larger volume. The concentrations of spiked controls determined by the two method were also highly correlated, although there was a significant bias (S7 Fig). We attribute this bias to differences in the calibration of Simoa and PCR, and possible differences in signal response in the two methods for synthetic calibrators of miRNA and the endogenous molecule.

## Conclusions

We have combined approaches to single molecule detection and specific labeling of single bases to develop a sensitive and specific assay for miRNA. The assay presented here offers complementary advantages to using PCR for detecting miRNA, namely, specificity to single base difference in sequence, simpler assay design via the use of a single probe, and avoiding the need for target extension for detection. The method also has the potential to address some of the intrinsic challenges of PCR, namely, amplification bias and carryover due to target amplification. At its current state of development, while being 600-fold times more sensitive that other bead-based assays, the sensitivity of the Simoa assay is, however, significantly lower than PCR. The potential to maximize the efficiency of capturing, labeling, and counting single molecules using Simoa, however, offers an attractive path to assays as sensitive as PCR.

The high specificity (>3 × 10^7^-fold) of the Simoa assay was achieved using just a single probe rather than multiple probes and primers used in most approaches for measuring miRNA. This high specificity resulted from the combination of the specificity of hybridization between the target and probe sequences, and the single base dynamic chemical incorporation of a label. The use of a single probe greatly simplifies the measurement of short sequences of NA, including simple probe design and assay development, and reducing the number of interactions that need to be screened for cross-reactivity in multiplex assays. The ability to detect single labels incorporated into the probe provides the high sensitivity of the assay. The use of encoded beads would also enable the measurement of multiple miRNA targets in the same array, as we have previously demonstrated for proteins.[22]

The Simoa assay enabled the measurement of miR-122 from the serum of patients with liver toxicity from acetaminophen. With development, this assay could have utility in clinical practice as it has the potential to deliver the user-independent sensitivity and time-to-result that are needed to inform clinical decision making. In terms of applications beyond liver toxicity, this assay offers a greatly simplified method for the early detection of more specific biomarkers in blood of patients with cancer (so called liquid biopsies), the early and rapid diagnosis of sepsis, pharmacokinetic measurements of interfering NA therapeutics, and the measurement of guide RNA used for gene editing systems, such as CRISPR/Cas9.

## Supporting information

S1 Fig Structure of amino-PEG linker of capture probe. (PDF)

S2 Fig Plot of Ct values determined using PCR as a function of [miR-122]. (PDF)

S3 Fig Specificity of Simoa assay for miR-122. (PDF)

S4 Fig Specificity of the PCR assay for miR-122. (PDF)

S5 Fig Ct values for patients and healthy controls. (PDF)

S6 Fig Bland-Altman plot for concentrations of miR-122 in serum samples of patients determined using Simoa and PCR. (PDF)

S7 Fig Correlation of concentration of synthetic miR-122 spiked into serum determined using Simoa and PCR. (PDF)

S1 Table Demographics and clinical chemistry results for patients with liver injury. (PDF)

S2 Table Sequences of nucleic acid molecules used to test the sensitivity and specificity of the Simoa assay. (PDF)

S3 Table Ct values determined using PCR as a function of [miR-122]. (PDF)

S4 Table AEB values for single base mismatch sequence. (PDF)

S5 Table Concentrations of miR-122 in samples using the Simoa assay. (PDF)

S6 Table Concentrations of miR-122 in samples using PCR. (PDF)
