# Supplementary figures and images for: Polymerase-free measurement of microRNA-122 with single base specificity using single molecule arrays: Detection of drug-induced liver injury
Source: PLoS One. 2017 Jul 5;12(7):e0179669. doi: 10.1371/journal.pone.0179669 (PMC5497960; doi:10.1371/journal.pone.0179669)

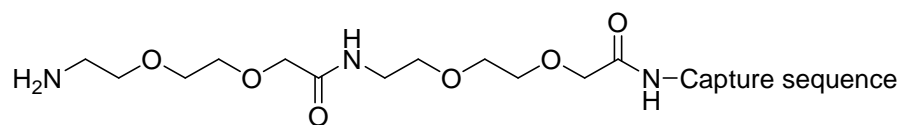

**S1 Figure.** Amino-PEG linker (“xx” in Table 1) located at the N-terminal end of the abasic PNA probe (**1**).

Supplement: S1 Fig — (PDF) [file pone.0179669.s001.pdf]

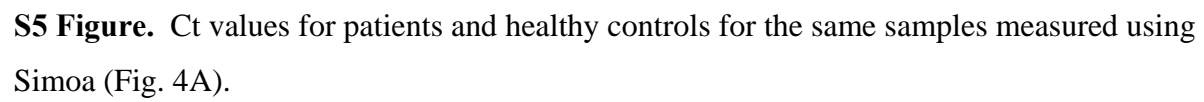

Supplement: S5 Fig — (PDF) [file pone.0179669.s005.pdf]
