# Supplementary material for: Polymerase-free measurement of microRNA-122 with single base specificity using single molecule arrays: Detection of drug-induced liver injury
Source: PLoS One. 2017 Jul 5;12(7):e0179669. doi: 10.1371/journal.pone.0179669 (PMC5497960; doi:10.1371/journal.pone.0179669)
Supplement: S2 Fig — (PDF) [file pone.0179669.s002.pdf]

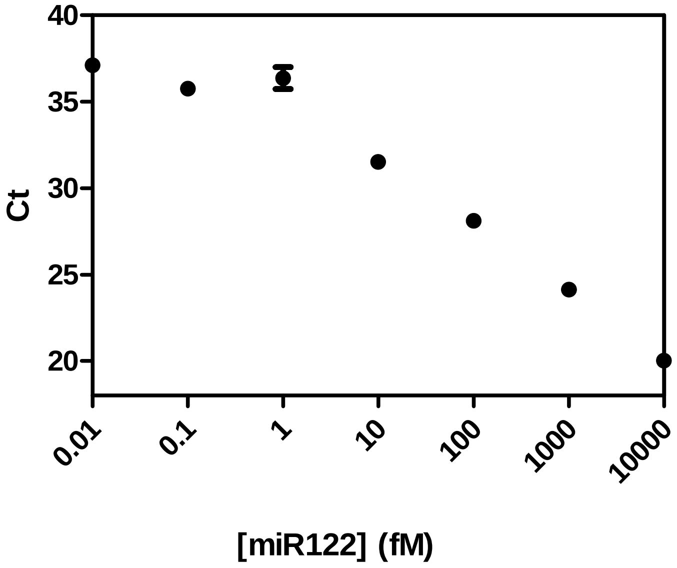

S2 Figure. Plot of Ct values determined using PCR as a function of the concentration of miR-122. Individual Ct values are provided in S3 Table. To estimate the limit of detection, the Ct values of the three lowest concentrations were averaged and the standard deviation determined. The Ct value at 3 s.d. below the mean Ct was 34, so the LOD was about 2.6 fM. Error bars are shown as 1 s.d.
