# Supplementary material for: Polymerase-free measurement of microRNA-122 with single base specificity using single molecule arrays: Detection of drug-induced liver injury
Source: PLoS One. 2017 Jul 5;12(7):e0179669. doi: 10.1371/journal.pone.0179669 (PMC5497960; doi:10.1371/journal.pone.0179669)
Supplement: S3 Fig — (PDF) [file pone.0179669.s003.pdf]

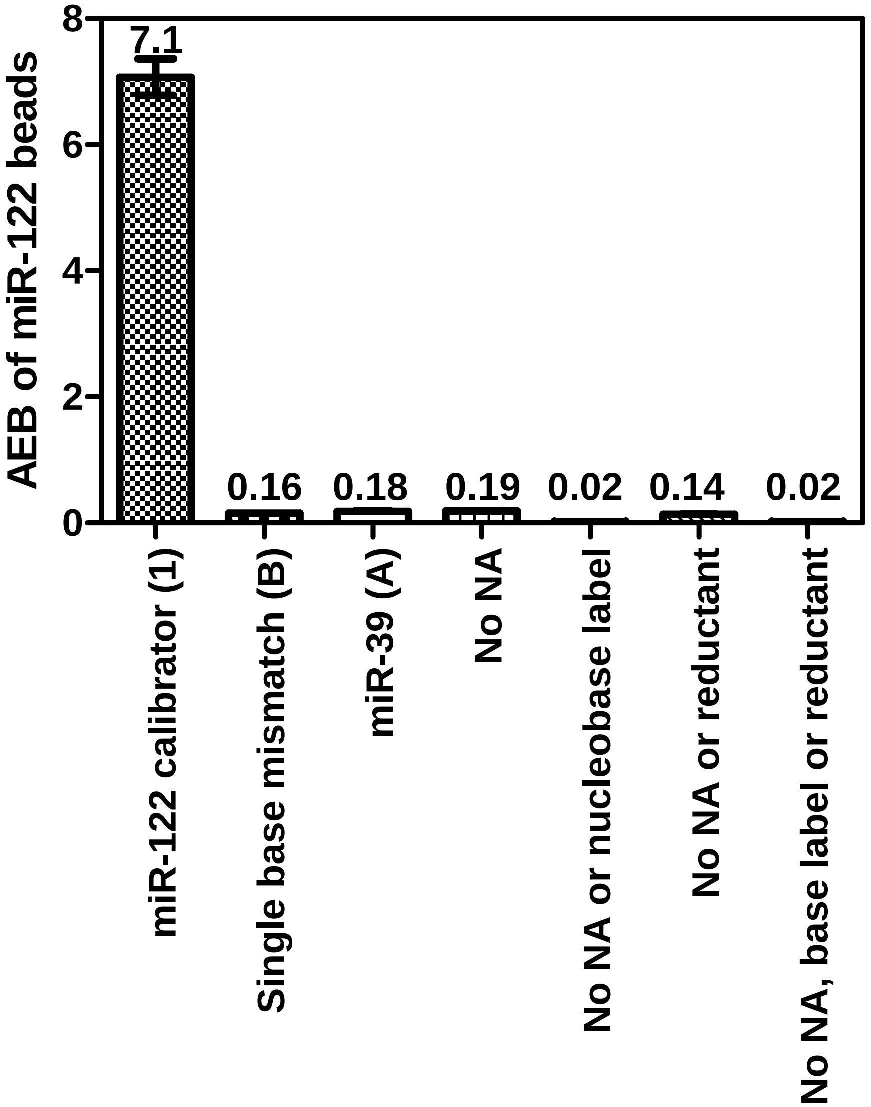

S3 Figure. Specificity of Simoa assay for miR-122. The chart shows AEB values of the miR-122 specific beads for: 15 nM of miR-122 calibrator (1st bar); 15 nM of miR-122 calibrator with a single base mismatch (2nd bar); 15 nM of miR-39 (3rd bar); no nucleic acid (4th bar); no nucleic acid and no nucleobase label (5th bar); no nucleic acid and no reductant (6th bar); and, no nucleic acid, no nucleobase, and no reductant (7th bar). The last 4 bars in the chart are reagent drop-out experiments that indicate the sources of background in the assay. These data indicate the non-specific binding of the nucleobase label to the beads dominates the background of the Simoa assay. Error bars are s.d. values from 2 replicates. Each bar is labelled with its AEB value.
