# Supplementary material for: Polymerase-free measurement of microRNA-122 with single base specificity using single molecule arrays: Detection of drug-induced liver injury
Source: PLoS One. 2017 Jul 5;12(7):e0179669. doi: 10.1371/journal.pone.0179669 (PMC5497960; doi:10.1371/journal.pone.0179669)
Supplement: S4 Fig — (PDF) [file pone.0179669.s004.pdf]

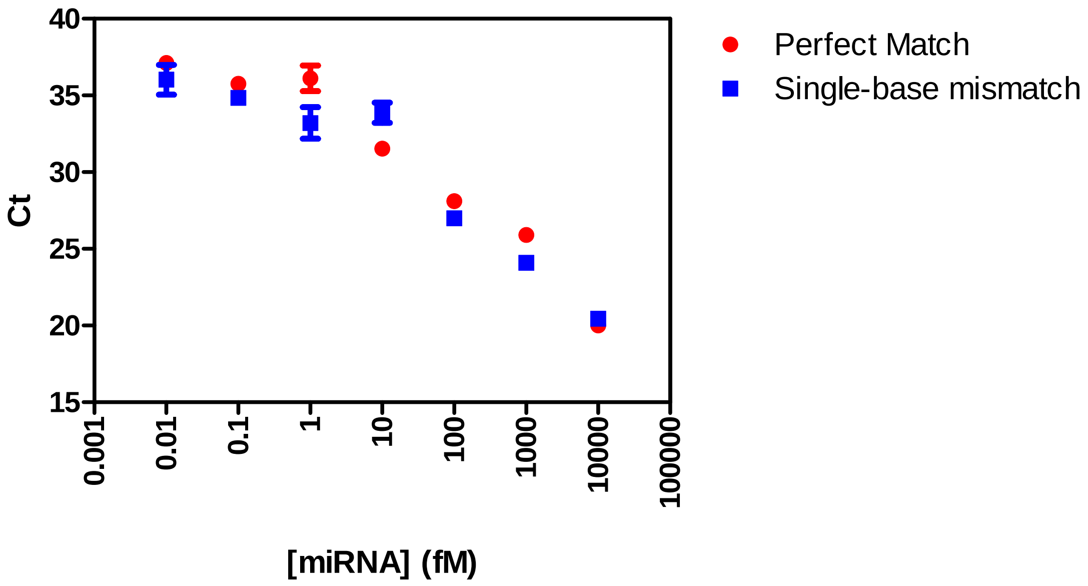

S4 Figure. Specificity of the PCR assay for miR-122. Plots of Ct values determined using PCR as a function of the concentration of miR-122 (red circles) and miR-122 with a single base mismatch at the 9th position (blue squares). Error bars are shown as 1 s.d. over triplicate PCR reactions from a single RT reaction.
