# Supplementary material for: Polymerase-free measurement of microRNA-122 with single base specificity using single molecule arrays: Detection of drug-induced liver injury
Source: PLoS One. 2017 Jul 5;12(7):e0179669. doi: 10.1371/journal.pone.0179669 (PMC5497960; doi:10.1371/journal.pone.0179669)
Supplement: S6 Fig — (PDF) [file pone.0179669.s006.pdf]

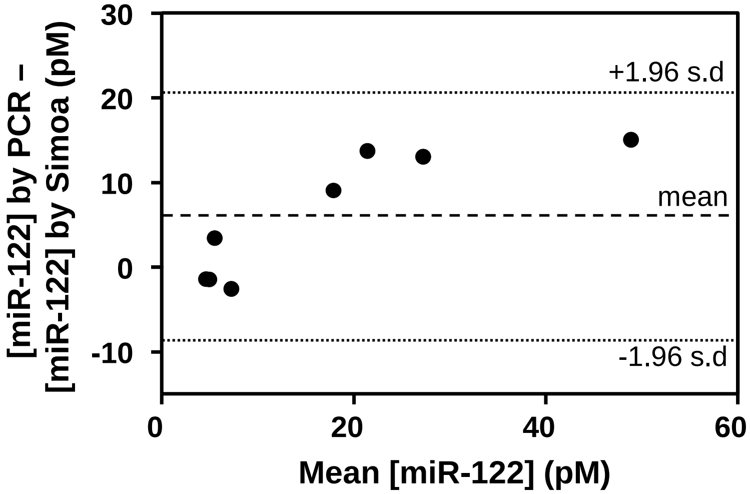

S6 Figure. Bland-Altman plot* for concentrations of miR-122 in serum samples of patients determined using Simoa and PCR. The dashed line is at the mean of the difference in concentration between the methods; the dotted lines are at ±1.96 s.d. of the mean.

*Bland JM, Altman DG. Statistical methods for assessing agreement between two methods of clinical measurement. Lancet. 1986 Feb 8;1(8476):307-10.
