# Supplementary material for: Polymerase-free measurement of microRNA-122 with single base specificity using single molecule arrays: Detection of drug-induced liver injury
Source: PLoS One. 2017 Jul 5;12(7):e0179669. doi: 10.1371/journal.pone.0179669 (PMC5497960; doi:10.1371/journal.pone.0179669)
Supplement: S7 Fig — (PDF) [file pone.0179669.s007.pdf]

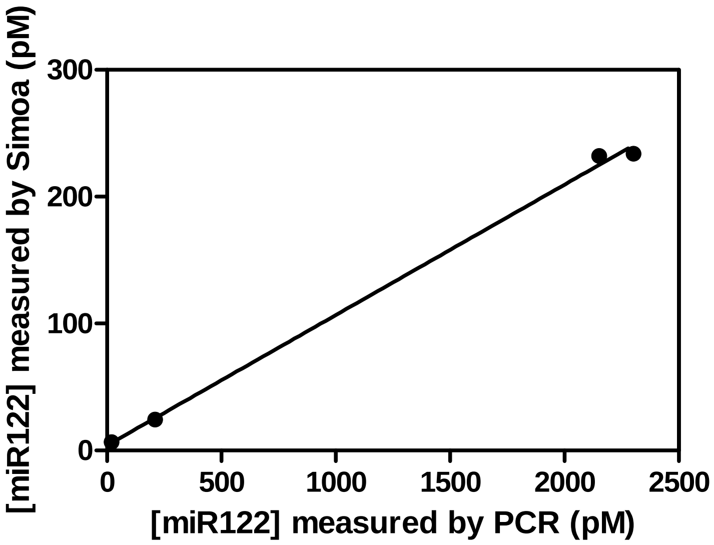


**S7 Figure.** Correlation of concentration of synthetic calibrator for miR-122 spiked into serum determined using Simoa and PCR ($r^2 = 0.998$).
