# Supplementary material for: Polymerase-free measurement of microRNA-122 with single base specificity using single molecule arrays: Detection of drug-induced liver injury
Source: PLoS One. 2017 Jul 5;12(7):e0179669. doi: 10.1371/journal.pone.0179669 (PMC5497960; doi:10.1371/journal.pone.0179669)
Supplement: S1 Table — (PDF) [file pone.0179669.s008.pdf]

S1 Table. Demographics and clinical chemistry results for patients with liver injury.

Patient ID	Age (yrs)	Sex	ALT activity (U/L) (ULN 50)	INR	Serum Creatinine (µmol/L)	ALP activity (U/L)	Bilirubin (µmol/dL)
**3**	56	M	2150	1.3	56	141	23
**4**	23	F	487	1.5	60	64	14
**6**	46	M	2963	1.4	62	139	23
**9**	63	F	726	1.2	68	169	14
