# Supplementary material for: Polymerase-free measurement of microRNA-122 with single base specificity using single molecule arrays: Detection of drug-induced liver injury
Source: PLoS One. 2017 Jul 5;12(7):e0179669. doi: 10.1371/journal.pone.0179669 (PMC5497960; doi:10.1371/journal.pone.0179669)
Supplement: S2 Table — (PDF) [file pone.0179669.s009.pdf]

S2 Table. Sequences of nucleic acid molecules used to test the sensitivity and specificity of the Simoa assay.

ID	Name	Sequence (5ˈ-3ˈ)
**A**	Calibrator for miR-122*	TGGAGTGT**G**ACAATGGTGTTTG
**B**	Calibrator for single base mismatch miR-122*	TGGAGTGT**T**ACAATGGTGTTTG
**C**	Off-target miR-39†	UCACCGGGUGUAAAUCAGCUUG

* We used DNA analogs as calibrators for measuring miRNA in the clinical samples. DNA enabled long term storage of calibrators and more reliable determination of miR-122 concentration in samples. We have previously shown that uracil and thymidine have similar efficiencies for specifically templating the dynamic covalent chemical reaction between aldehyde-modified cytosine and secondary amine of the abasic PNA probe upon duplex formation.[⊥] We have also shown equivalent performance of RNA and DNA calibrators for miR-122 in the assay presented here (data not shown).

†miRNeasy Serum/Plasma Spike-In Control (Cat No./ID:219610, Qiagen):10 pmol lyophilized *C.elegans* miR-39 miRNA mimic.

[⊥]Bowler FR. Ph.D. Dissertation, University of Edinburgh, UK, 2011.
