# Supplementary material for: Polymerase-free measurement of microRNA-122 with single base specificity using single molecule arrays: Detection of drug-induced liver injury
Source: PLoS One. 2017 Jul 5;12(7):e0179669. doi: 10.1371/journal.pone.0179669 (PMC5497960; doi:10.1371/journal.pone.0179669)
Supplement: S3 Table — (PDF) [file pone.0179669.s010.pdf]

S3 Table. Ct values determined using PCR as a function of the concentration of miR-122. The concentration is that of miR-122 in the reverse transcription (RT) reaction in the process for testing samples described in the Materials and Methods section. One RT reaction was carried out, and PCR performed on three aliquots from this reaction solution. n.d. = not determined.

Concentration (fM)	Ct values			Mean Ct	SD	CV
**0**	37.10841	n.d.	n.d.	37.108	n.d	n.d
**0.1**	35.75835	n.d.	n.d.	35.758	n.d	n.d
**1**	35.65074	36.61235	36.83607	36.366	0.630	1.7%
**10**	31.58425	31.55038	31.44276	31.526	0.074	0.2%
**100**	28.31135	27.97488	28.05834	28.115	0.175	0.6%
**1,000**	24.08295	24.15437	24.16391	24.134	0.044	0.2%
**10,000**	19.94744	19.8787	20.23545	20.021	0.189	0.9%
