# Supplementary material for: Polymerase-free measurement of microRNA-122 with single base specificity using single molecule arrays: Detection of drug-induced liver injury
Source: PLoS One. 2017 Jul 5;12(7):e0179669. doi: 10.1371/journal.pone.0179669 (PMC5497960; doi:10.1371/journal.pone.0179669)
Supplement: S4 Table — (PDF) [file pone.0179669.s011.pdf]

S4 Table. AEB values for single base mismatch sequence (Molecule **B** in S2 Table) from 0 pM to 15 μM. No increase in signal above background was observed across the concentration range.

Concentration of mismatched target (B)	Mean AEB	SD	CV	Signal-to-background ratio
15 μM	0.086	0.002	2%	0.92
1.5 μM	0.071	0.004	6%	0.76
150 nM	0.079	0.016	20%	0.84
15 nM	0.083	0.014	17%	0.89
1.5 nM	0.091	0.009	10%	0.98
150 pM	0.093	0.007	7%	1.00
15 pM	0.089	0.004	4%	0.96
0 pM (background)	0.093	0.009	9%	1.00
