# Supplementary material for: Polymerase-free measurement of microRNA-122 with single base specificity using single molecule arrays: Detection of drug-induced liver injury
Source: PLoS One. 2017 Jul 5;12(7):e0179669. doi: 10.1371/journal.pone.0179669 (PMC5497960; doi:10.1371/journal.pone.0179669)
Supplement: S5 Table — (PDF) [file pone.0179669.s012.pdf]

S5 Table. Concentration of miR-122 in clinical samples and serum control samples spiked with synthetic miR-122 determined using Simoa.

Patient ID	Sample number*	Sample volume (μL)	Concentration in 50 μL reaction (pM)	Recovery (%)†	Concentration in sample (pM)	Spiked concentration
**HV1**	1	90	1.51	51	*2.65*‡	
	2	90	3.91	81	*4.27*‡	
**HV2**	1	95	1.51	84	*1.51*‡	
	2	95	3.81	94	*3.41*‡	
**HV3**	1	95	1.34	83	*1.37*‡	
	2	95	2.95	84	*2.97*‡	
**HV4**	1	100	1.13	78	*1.17*‡	
	2	100	3.32	70	*3.81*‡	
**3**	1	105	2.85	41	5.28	
	2	105	6.46	58	8.54	
**4**	1	95	11.57	73	13.34	
	2	95	13.66	79	14.56	
**6**	1	95	19.67	80	20.71	
	2	95	31.43	64	41.35	
**9**	1	90	3.21	75	3.81	
	2	90	6.25	98	5.67	
**Controls**						
**1**	1	100	n.d.	49	n.d.	10000
**2**	2	100	n.d.	67	n.d.	10000
**3**	3	100	162.66	56	232.13	1000
**4**	4	100	126.47	43	233.96	1000
**7**	5	100	13.78	45	24.31	100
**8**	6	100	3.69	44	6.70	10

n.d. = not determined

*For each patient and healthy volunteer, miRNA was extracted from two samples and its concentration was determined.

† Recovery of miRNA from the sample was determined by PCR using miR-39 control spikes. The concentration in samples were corrected for this recovery.

‡ The AEB values determined for these samples were close together and slightly offset from the background AEB (Fig. 4A). This observation could be explained by a systematic difference in the background signal of the sample matrix compared to the calibration buffer, rather than by specific measurement of miR-122. Without further experimental confirmation, we regard these samples as not quantified for miR-122.
