# Supplementary material for: Polymerase-free measurement of microRNA-122 with single base specificity using single molecule arrays: Detection of drug-induced liver injury
Source: PLoS One. 2017 Jul 5;12(7):e0179669. doi: 10.1371/journal.pone.0179669 (PMC5497960; doi:10.1371/journal.pone.0179669)
Supplement: S6 Table — (PDF) [file pone.0179669.s013.pdf]

S6 Table. Concentration of miR-122 in clinical samples and serum control samples spiked with synthetic miR-122 determined using PCR.

Patient ID	Sample number*	Volume sample (μL)	Ct	Copies/μL in PCR reaction	Copies in sample	Recovery (%)	Concentration in sample (pM)	Spiked concentration
**HV1**	1	90	34.4	76	3,345,710	51	*0.12*‡	
	2	90	34.1	92	4,050,941	81	*0.09*‡	
**HV2**	1	95	32.4	281	12,355,108	84	*0.26*‡	
	2	95	32.9	200	8,783,626	94	*0.16*‡	
**HV3**	1	95	33.3	156	6,875,551	83	*0.15*‡	
	2	95	33.4	147	6,461,253	84	*0.13*‡	
**HV4**	1	100	33.2	169	7,419,621	78	*0.16*‡	
	2	100	33.4	152	6,705,783	70	*0.16*‡	
**3**	1	105	29.1	2,295	100,969,205	41	3.88	
	2	105	27.9	4,942	217,428,017	58	5.96	
**4**	1	95	25.6	21,300	937,196,939	73	22.41	
	2	95	25.1	29,113	1,280,986,047	79	28.31	
**6**	1	95	24.8	35,139	1,546,107,754	80	33.74	
	2	95	24.3	47,006	2,068,243,613	64	56.41	
**9**	1	90	27.4	6,702	294,868,894	75	7.24	
	2	90	27.8	5,120	225,300,348	98	4.24	
**Controls**								
**1**	1	100	14.5	23,245,908	1,022,819,938,047	49	34380.66	10000
**2**	2	100	14.5	23,790,920	1,046,800,483,612	67	25999.14	10000
**3**	3	100	18.7	1,653,095	72,736,170,424	56	2151.74	1000
**4**	4	100	19.0	1,363,925	60,012,704,821	43	2301.44	1000
**7**	5	100	22.7	130,673	5,749,590,955	45	210.31	100
**8**	6	100	26.4	12,485	549,318,183	44	20.67	10

*For each patient and healthy volunteer, miRNA was extracted from two samples and its concentration was determined.

† Recovery of miRNA from the sample was determined by PCR using miR-39 control spikes. The concentration in samples were corrected for this recovery.

‡ The Ct values of samples from healthy volunteers were above or slightly below those of background samples (S3 Table), so the detection of miR-122 in these samples is ambiguous.
